# Transcutaneous auricular vagus nerve stimulation for disorders of consciousness: a narrative review of neurophysiological mechanisms, clinical evidence, and future directions

**DOI:** 10.3389/fnhum.2026.1837913

**Published:** 2026-08-05

**Authors:** Shutong Mei, Guan Fu, Ruixue Gao, Wei Li

**Affiliations:** 1 School of Special Education and Rehabilitation, Shandong Medical and Pharmaceutical University, Yantai, Shandong, China; 2 Department of Rehabilitation Medicine, Shandong Medical and Pharmaceutical University Hospital, Binzhou, Shandong, China

**Keywords:** consciousness recovery, disorders of consciousness, neuromodulation, neurophysiological mechanisms, transcutaneous auricular vagus nerve stimulation

## Abstract

Disorders of consciousness (DoC) following severe brain injury remain difficult to treat, and effective non-invasive therapeutic options are still limited. Transcutaneous auricular vagus nerve stimulation (taVNS) has recently emerged as a promising neuromodulation approach in this field. In this narrative review, we synthesize recent evidence on taVNS for DoC across four domains: neurophysiological mechanisms, multimodal monitoring evidence, clinical efficacy, and stimulation parameter optimization with safety considerations. Current mechanistic models suggest that taVNS may influence consciousness through the auricular branch of the vagus nerve, engaging brainstem nuclei such as the nucleus tractus solitarius and locus coeruleus, and subsequently modulating thalamo-cortical and large-scale cortical networks; this remains a biologically plausible working model rather than a validated causal pathway. Preliminary clinical data suggest that patients in a minimally conscious state may respond more often than those with unresponsive wakefulness syndrome/vegetative state; however, this is a subgroup-level signal—the only sham-controlled randomized trial to date found no significant overall between-group difference—and the evidence remains limited by small samples, protocol heterogeneity, and short follow-up. Multimodal tools including electroencephalography, functional near-infrared spectroscopy, heart rate variability, and serum biomarkers may improve mechanistic understanding and responder stratification, but integrated evidence within the same patient cohorts remains scarce. Overall, taVNS appears to be a promising adjunctive intervention for DoC, but its routine clinical application remains provisional until ongoing multicenter randomized trials clarify efficacy, durability, optimal stimulation parameters, and responder profiles.

## 1 Introduction

Disorders of consciousness (DoC) represent a pathological state of impaired wakefulness and awareness caused by severe brain injury. Based on the level of consciousness, patients can be classified into coma, vegetative state (VS)/unresponsive wakefulness syndrome (UWS), and minimally conscious state (MCS). Prolonged disorders of consciousness (pDoC) refers to DoC persisting for more than 28 days (Golden et al., 2024). In China, with advances in emergency medicine and intensive care technology, an increasing number of patients with severe traumatic brain injury survive; however, the number of DoC patients continues to rise annually. These patients require long-term care and rehabilitation, imposing a heavy burden on families and society, which underscores the urgent need for high-quality clinical research and standardized diagnosis and treatment.

Precise diagnosis of DoC patients is extremely challenging, as no single assessment method can provide a definitive diagnosis. The Coma Recovery Scale-Revised (CRS-R) is currently the recommended standardized tool for assessing the level of consciousness (Di et al., 2017). Advanced technologies such as electroencephalography (EEG), functional magnetic resonance imaging (fMRI), and positron emission tomography (PET) are also used to measure consciousness levels in DoC patients, but no single technology can independently determine the level of consciousness.

Currently, treatment options for pDoC are very limited. Amantadine is the only consciousness-promoting drug supported by evidence-based guidelines, but the strongest recommendation applies to a restricted subgroup—adults with traumatic VS/UWS or MCS who are 4–16 weeks post-injury, in whom amantadine (100–200 mg twice daily) may accelerate early functional recovery; this recommendation should not be generalized to all etiologies or all stages of DoC (Giacino et al., 2018). Deep brain stimulation (DBS) has shown some efficacy in individual cases, but its invasive nature and high treatment costs limit clinical application (Raguž et al., 2021). Therefore, exploring non-invasive treatments that are safe, effective, and easy to implement holds significant clinical importance.

Vagus nerve stimulation (VNS) refers to a family of neuromodulation approaches that differ in invasiveness, device type, and regulatory indication, and these should not be conflated. Implanted cervical VNS, which delivers current to the cervical vagus nerve through surgically placed electrodes and a pulse generator, is approved by the United States Food and Drug Administration (FDA) for drug-resistant epilepsy and treatment-resistant depression (Giordano et al., 2017; Milby et al., 2008). A separate implanted vagal blocking device is approved for weight management in obesity, whereas non-invasive approaches—including transcutaneous cervical VNS and transcutaneous auricular vagus nerve stimulation (taVNS)—have been cleared or studied for indications such as cluster headache and migraine and explored in stroke rehabilitation (Austelle et al., 2022; Hilz, 2022). Compared with implanted VNS, taVNS offers significant advantages: no surgery required, low cost, simple operation, good repeatability, and high patient compliance, making it particularly suitable for pDoC patients requiring long-term treatment.

Although previous reviews have elaborated on the animal experimental basis of taVNS, the translation of their conclusions to clinical practice has long been limited by small sample sizes, high design heterogeneity, and the lack of objective neurophysiological evidence in human studies (Wang et al., 2023). Recently, published sham-controlled reports, longitudinal case observations, bedside neurophysiological studies, and multicenter trial protocols have suggested that taVNS research in pDoC is moving from predominantly exploratory work toward more clinically oriented investigation. This review aims to synthesize the latest research progress, focusing on the neurophysiological mechanisms of taVNS, evaluating its neurophysiological evidence from a multimodal perspective, reviewing the clinical application evolution of taVNS in DoC treatment, and discussing strategies for stimulation-parameter optimization and safety assessment. Throughout the review, completed clinical studies are distinguished from case reports and protocols so that preliminary signals are not overstated as established efficacy.

This article is a narrative review rather than a formal systematic review or meta-analysis. Our aim was to provide a clinically oriented synthesis of recent mechanistic, neurophysiological, and clinical evidence on taVNS for disorders of consciousness. The literature search was updated close to the date of revision, and the body of evidence considered spans studies published from 2017 to 2026.

Relevant English-language literature was identified through searches of PubMed, Web of Science, and Embase, from database inception to June 30, 2026, using combinations of terms related to “transcutaneous auricular vagus nerve stimulation,” “transauricular vagus nerve stimulation,” “vagus nerve stimulation,” “disorders of consciousness,” “prolonged disorders of consciousness,” “minimally conscious state,” and “unresponsive wakefulness syndrome/vegetative state.” The core search string was: (“transcutaneous auricular vagus nerve stimulation” OR “transauricular vagus nerve stimulation” OR “vagus nerve stimulation” OR taVNS OR tVNS) AND (“disorders of consciousness” OR “prolonged disorders of consciousness” OR “minimally conscious state” OR “vegetative state” OR “unresponsive wakefulness syndrome” OR coma); database-specific syntax was adapted as appropriate. Only English-language records were included. Eligible reports comprised human studies of taVNS or closely related vagal stimulation in patients with disorders of consciousness, together with mechanistic studies directly relevant to consciousness recovery. Completed clinical studies were considered separately from single-case reports, observational/methodological reports, and published trial protocols, and these categories were not treated as equivalent sources of clinical-effect evidence.

Given the narrative nature of this review, formal risk-of-bias assessment and quantitative evidence synthesis were not performed; instead, we prioritized completed clinical studies, mechanistic studies directly relevant to consciousness recovery, and recent trial protocols likely to shape the development of the field. Relative to the mechanistic and systematic reviews published in 2023 (Wang et al., 2023), the specific contribution of the present review is to integrate four strands of evidence—neurophysiological mechanisms, multimodal monitoring, clinical efficacy, and stimulation-parameter optimization with safety—within a single clinically oriented framework, while explicitly distinguishing direct evidence in DoC from indirect, cross-population, and hypothetical evidence. More recently, a broader 2026 narrative review from our group surveyed non-invasive and minimally invasive neuromodulation for DoC across multiple modalities—transcranial magnetic stimulation, transcranial electrical stimulation, median nerve stimulation, taVNS, transcranial focused ultrasound, acupuncture, and spinal cord stimulation—and concluded that current evidence is comparatively strongest for high-frequency repetitive TMS, right median nerve stimulation, and spinal cord stimulation (Cao et al., 2026). The present review is deliberately narrower and deeper: rather than comparing modalities, it focuses specifically on taVNS and integrates its neurophysiological mechanisms, multimodal monitoring evidence, completed trials and registered protocols, and stimulation-parameter optimization with safety within a single evidence-tiered framework, thereby providing a more granular, taVNS-specific synthesis intended to inform standardized application and future trial design.

## 2 Principles and mechanisms of taVNS

**Figure 1:**
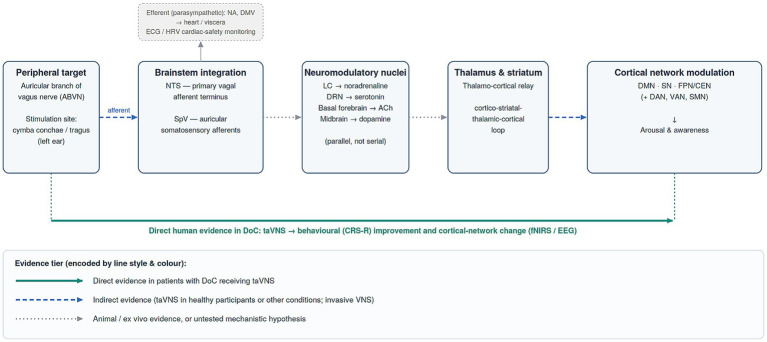
Evidence-tiered working model for taVNS in disorders of consciousness. Solid green lines indicate direct evidence from patients with DoC receiving taVNS; dashed blue lines indicate indirect evidence from healthy participants, other clinical populations, or invasive VNS; dotted gray lines indicate animal/ex vivo evidence or untested mechanistic hypotheses. The diagram is intended to summarize evidence tiers rather than to establish a validated causal sequence.

The mechanisms by which taVNS may facilitate recovery of consciousness are best understood as a working neurobiological model rather than a fully validated pathway in DoC. Current evidence supports a plausible “peripheral stimulation target → brainstem integration center → cortical network modulation” cascade, but direct confirmation of each step in DoC populations remains incomplete. To avoid overstating the strength of this model, the supporting evidence in the following sections can be read as a hierarchy of decreasing directness: (i) direct evidence from patients with DoC receiving taVNS; (ii) indirect evidence from taVNS studies in healthy participants or other clinical populations; (iii) evidence from invasive VNS; (iv) evidence from animal or ex vivo studies; and (v) mechanistic hypotheses that remain untested in DoC. Several pathways proposed below, including brainstem monoaminergic modulation and large-scale network reconfiguration, are supported to different degrees across these tiers. Figure 1 summarizes this framework and distinguishes the tiers of supporting evidence by line style and colour.

### 2.1 Peripheral stimulation target: anatomical basis of the auricular branch of the vagus nerve

The vagus nerve is the tenth cranial nerve, with its fiber composition predominantly consisting of sensory afferent fibers (approximately 80%) and relatively fewer motor efferent fibers (approximately 20%). The sensory innervation of the human external ear is complex, involving the auricular branch of the vagus nerve (ABVN), auriculotemporal nerve, great auricular nerve, and lesser occipital nerve, among others (Peuker and Filler, 2002). Anatomical studies have confirmed that the tragus, cymba conchae, and cavum conchae regions contain cutaneous afferent fibers of the vagus nerve. Notably, the concha region is the only body surface area with extensive ABVN distribution, with a distribution density approaching 100%, making it an ideal target for transcutaneous vagus nerve stimulation (Yakunina et al., 2017).

The ABVN primarily comprises myelinated Aβ fibers, myelinated Aδ fibers, and unmyelinated C fibers (Bermejo et al., 2017). The number of Aβ fibers in the bilateral ABVN is essentially symmetrical (Safi et al., 2016). Due to significant individual variability in ABVN fiber quantity, determining optimal stimulation sites and parameters for taVNS remains challenging. Currently, detailed anatomical studies of nerve fiber types within the ABVN remain insufficient, particularly lacking data suitable for computational modeling.

Although the anatomical distribution of the auricular branch of the vagus nerve provides a rational peripheral target for taVNS, individual variability in fiber composition and limited histological detail mean that the relationship between stimulation site, fiber recruitment, and downstream clinical response remains insufficiently defined.

### 2.2 Brainstem integration center: NTS–LC projection pathway

taVNS is thought to stimulate the auricular branch of the vagus nerve, whose afferent signals may ascend to the brainstem and engage several nuclei, including the nucleus tractus solitarius (NTS), dorsal motor nucleus of the vagus, nucleus ambiguus, and spinal trigeminal nucleus. Among these structures, the NTS is widely regarded as the most plausible central relay for the afferent effects of taVNS. However, direct evidence confirming this pathway specifically in patients with DoC remains limited.

taVNS stimulates the ABVN, whose afferent signals ascend along the vagus nerve to the brainstem. The central afferent input from the ABVN principally terminates in the nucleus tractus solitarius (NTS), the primary termination nucleus for vagal sensory afferents, while somatosensory components of the auricular region project to the spinal trigeminal nucleus (SpV) (Wang et al., 2014; Neuhuber and Berthoud, 2021). By contrast, the nucleus ambiguus (NA) and the dorsal motor nucleus of the vagus (DMV) are predominantly efferent (output) nuclei that mediate the “descending” parasympathetic effects of the vagus nerve and are not primary afferent termination sites (Berthoud and Neuhuber, 2000). The SpV mainly processes pain, temperature, and tactile information from the face and head–neck region, and there is currently no direct evidence that it participates in taVNS-mediated consciousness modulation. The NTS therefore serves as the critical relay between the vagus nerve and the central nervous system (CNS), receiving and integrating afferent information before projecting to the upper brainstem and cortex, and is considered the core hub for the central effects of taVNS.

Afferent signals received by the NTS can be relayed, in part via the ascending reticular activating system (ARAS), to several neuromodulatory nuclei. These include the locus coeruleus (LC), the principal source of cortical norepinephrine (NE); the dorsal raphe nucleus (DRN), a major source of serotonin; and, through polysynaptic connections, the basal forebrain cholinergic system that supplies acetylcholine (ACh), together with midbrain dopaminergic influences. The degree to which each of these systems is engaged by taVNS in DoC remains incompletely characterized, and the relevant evidence differs across neurotransmitter systems (Breit et al., 2018; Hulsey et al., 2017; Zhang et al., 2019).

The central projections of the ABVN overlap substantially with the classical central vagal pathway, reaching the NTS, LC, and related nuclei (Butt et al., 2020; Knowles and Aziz, 2009); rather than acting as a single serial chain, these nuclei modulate multiple downstream targets in parallel through distinct neurotransmitter systems. LC activation releases NE, which contributes to the regulation of alertness, attention, stress responses, and sleep–wake cycles (Hulsey et al., 2017; Farrand et al., 2023). DRN activation releases serotonin, which has been linked to connectivity within the default mode network (DMN). The cholinergic system is a key component of cortical arousal, and VNS has been shown to induce cerebral ACh release (Meyers et al., 2019). The release of these neurotransmitters can broadly engage neuromodulatory systems and promote neuroplasticity (Hays et al., 2013), which together support wakefulness and alertness and provide a neurobiological rationale for exploring the therapeutic potential of taVNS in modulating consciousness.

### 2.3 Cascading activation of the thalamo-cortical network

The LC and DRN project to the thalamus through ascending pathways. As a “relay gateway” for subcortical–cortical information transfer, thalamic activation may enhance the efficiency of thalamo-cortical transmission and, together with striatal involvement, has been conceptualized within a “cortical–striatal–thalamic–cortical” circuit model. At the cortical level, the effects of taVNS are commonly discussed in relation to several large-scale functional networks. Rather than a fixed set of “consciousness networks,” these are better viewed as an organizing framework and include the default mode network (DMN), salience network (SN), frontoparietal network (FPN)/central executive network (CEN), dorsal attention network (DAN), ventral attention network (VAN), and sensorimotor network (SMN; Uddin et al., 2019). The FPN and CEN are related but not strictly interchangeable constructs, and these networks show varying degrees of impairment in DoC. Among them, the DMN, SN, and FPN/CEN are most frequently discussed in relation to consciousness modulation and have comparatively more taVNS-related evidence.

The DMN is commonly implicated in introspective thinking and self-referential cognitive activities, with key nodes including the medial prefrontal cortex (mPFC) and posterior cingulate cortex/precuneus. Indirect evidence from non-DoC taVNS studies suggests that serotonergic and related neuromodulatory systems may influence DMN activity and connectivity; however, whether this mechanism improves internal awareness in DoC patients remains unproven and should be treated as a mechanistic hypothesis rather than direct clinical evidence (Rong et al., 2016).

The SN is involved in detecting salient internal and external stimuli and in coordinating transitions between internally oriented and externally oriented processing, with key regions including the anterior cingulate cortex and insula (Menon and Uddin, 2010). LC-noradrenergic activity is plausibly relevant to salience detection and arousal, but direct evidence that taVNS-driven LC activation improves salience-network function or behavioral responsiveness in DoC is still limited. Therefore, SN modulation should be described as a candidate pathway requiring direct validation in DoC cohorts rather than as an established therapeutic mechanism.

Evidence on frequency-dependent network effects comes largely from other clinical populations and should be regarded as indirect. In studies of patients with major depression or migraine, functional connectivity between the DMN and CEN appeared to change with taVNS frequency—1 Hz stimulation was associated with weakened DMN–CEN connectivity and 25 Hz with enhanced connectivity, while both frequencies were associated with changes in DMN–SN connectivity (Yakunina et al., 2017; Rong et al., 2016; Li et al., 2016). Because these findings were not obtained in DoC cohorts and did not constitute a direct head-to-head comparison of 1 Hz versus 25 Hz in DoC, they are best interpreted as cross-disorder, hypothesis-generating evidence rather than as a direct explanation of treatment responses in DoC.

In summary, taVNS may influence wakefulness and consciousness-related processing through bottom-up vagal afferent pathways, brainstem neuromodulatory systems, thalamo-cortical circuits, and large-scale cortical networks (Hilz, 2022). Compared with invasive neuromodulation, taVNS avoids surgical implantation and is therefore attractive for long-term rehabilitation research in pDoC; however, its network-level effects remain incompletely validated and should not be described as specific or established circuit repair (Wu et al., 2021).

## 3 Multimodal evidence for taVNS treatment of DoC

Previous reviews have extensively summarized anti-inflammatory and anti-apoptotic mechanisms of taVNS in animal models (Wang et al., 2023). In contrast, recent human studies have increasingly focused on brain-network modulation and bedside neurophysiological monitoring. These developments provide clinically relevant signals, although the evidence remains heterogeneous and is not yet sufficient to establish a unified mechanistic framework. In the sections below, multimodal measures are classified according to their main evidentiary role: behavioral scales primarily define diagnostic and clinical-response endpoints; fNIRS, EEG, fMRI, PET, and HRV may support phenotyping or longitudinal treatment-effect monitoring depending on study design; and serum NSE, NfL, and S100B remain exploratory candidate biomarkers rather than validated indicators of taVNS response.

### 3.1 Behavioral assessment scales

Behavioral assessment scales remain the foundational tools for efficacy evaluation in clinical studies. The CRS-R provides a standardized instrument for quantifying consciousness through systematic scoring across six subscales—auditory, visual, motor, oromotor/verbal, communication, and arousal (Di et al., 2017). Although the CRS-R remains the reference standard for behavioral diagnosis, recent larger RCTs and protocols (Wang et al., 2024) have incorporated supplementary measures: the Full Outline of UnResponsiveness (FOUR) scale for evaluating brainstem reflexes and respiratory patterns in intubated or aphasic patients, and the Extended Glasgow Outcome Scale (GOS-E) for functional outcome during long-term follow-up. In addition, the Nociception Coma Scale-Revised (NCS-R) has been explored as an investigational aid for setting stimulation intensity in non-communicative patients; importantly, the NCS-R indexes behavioral responses to nociceptive stimulation rather than effective vagal-fiber recruitment.

Throughout this review, “treatment response” is not used as a single undifferentiated category. Where possible, we distinguish between a change in the total CRS-R score, the emergence of a new diagnostically relevant behavior, a diagnostic transition from VS/UWS to MCS or from MCS− to MCS+, emergence from MCS (EMCS), and improvement in longer-term functional outcomes. These endpoints carry different clinical weight and should not be treated as interchangeable evidence of effectiveness.

### 3.2 Neuroimaging evidence

#### 3.2.1 fNIRS

As an emerging bedside neural function monitoring tool, fNIRS technology offers advantages of portability, non-invasiveness, and real-time monitoring, demonstrating application potential in revealing the neural effects of taVNS and its impact on consciousness recovery (Si et al., 2018; Zhang et al., 2018). As a mechanistic research tool, fNIRS can intuitively present the modulatory effects of taVNS on cerebral hemodynamics and network connectivity. As an efficacy assessment tool, compared with fMRI, fNIRS does not require patient transport and can perform bedside long-term dynamic monitoring, making it particularly suitable for evaluating longitudinal changes in brain functional connectivity during taVNS treatment. Gao et al. (2025) used fNIRS to monitor cerebral hemodynamic changes in a UWS patient receiving taVNS combined with patient-preferred music therapy. After 3 months of treatment, functional connectivity between the left dorsolateral prefrontal cortex and prefrontal regions increased from 0.06 to 0.55, in parallel with a clinical transition from UWS to MCS+. As this observation derives from a single case and from a combined intervention design, it should be interpreted as preliminary support for the feasibility of fNIRS-based monitoring rather than definitive validation of a specific taVNS mechanism or “external consciousness network” pathway. Despite its technical advantages, the measurement reliability and clinical value of fNIRS in DoC still require further validation in larger cohorts.

#### 3.2.2 fMRI

fMRI technology can track changes in brain network connectivity patterns in DoC patients, particularly functional changes in the DMN and other core neural networks before and after taVNS intervention, which may reflect a potential for functional network remodeling; such changes are associative and should not be equated with confirmed neural-circuit repairs (Kondziella et al., 2020; Snider and Edlow, 2020). By detecting blood-oxygen-level-dependent (BOLD) signal changes and cerebral blood flow, fMRI can reveal the modulatory effects of taVNS on brain functional networks with relatively high spatial resolution. In healthy populations, studies have confirmed that taVNS can activate key brain regions of the vagal afferent pathway, including the NTS, LC, insula, anterior cingulate cortex, frontal cortex, and cerebellum, with activation patterns broadly consistent with the neurophysiological characteristics of invasive VNS (Badran et al., 2018). However, current fMRI studies of taVNS in DoC remain predominantly small-sample exploratory designs, and practical issues such as high equipment costs and the need to transport patients to imaging centers limit its application in bedside long-term monitoring. Future studies combining resting-state and task-based fMRI with longitudinal designs may help clarify whether taVNS is associated with dynamic remodeling of consciousness-related networks in DoC. At present, however, fMRI evidence in DoC remains limited and should be considered exploratory.

#### 3.2.3 PET

PET imaging can evaluate cerebral metabolic levels and consciousness-related network activity in DoC patients, with demonstrated diagnostic superiority over other imaging modalities. Stender et al. (2014) reported that ^18^F-FDG PET achieved 93% diagnostic sensitivity for MCS in 126 DoC patients, notably identifying consciousness-compatible metabolic patterns in 32% of behaviorally diagnosed UWS patients. This ability to detect residual consciousness that may be underestimated by behavioral assessment suggests that PET could help refine patient stratification and identify potential taVNS responders. However, direct PET evidence on taVNS effects in DoC remains scarce. At present, PET should be viewed mainly as a promising phenotyping and responder-selection tool rather than a validated modality for monitoring taVNS efficacy.

### 3.3 Neurophysiological evidence

EEG technology can capture the dynamic evolution of brain electrical activity before and after taVNS intervention in real time, but EEG monitoring in DoC patients often faces technical challenges such as severe artifact interference and insufficient standardization of data interpretation (Kondziella et al., 2020). EEG-derived biomarkers, including background EEG activity, reactivity features, event-related potentials (ERPs), and spectral power distribution, play crucial roles in DoC diagnosis and prognosis assessment (Edlow et al., 2021). Among these, the preservation of reactive alpha rhythms reflects the integrity of neural information processing and the functional state of thalamo-cortical circuits (Porcaro et al., 2021). The reactivity characteristics of alpha rhythms also aid in differentiating DoC subtypes: MCS patients tend to exhibit more abundant alpha wave activity compared to VS/UWS patients (Bai et al., 2021). Additionally, EEG ERPs, particularly the P300 component, can serve as electrophysiological windows reflecting residual cognitive processing capacity in DoC patients (Rollnik, 2019). Current research indicates that EEG application has evolved from simple background rhythm analysis to ERP and network connectivity analysis. López-Rodríguez et al. (2025) described a standardized approach for combining taVNS with EEG assessment, including ERP-based markers such as MMN and P300, in DoC research. Because this report is primarily methodological and appears related to a previously reported clinical cohort, it should be cited as support for EEG-assessment feasibility rather than as an independent source of treatment-efficacy evidence.

### 3.4 Serum neural injury biomarkers

Beyond traditional imaging indicators, recent research has begun to consider objective fluid and physiological biomarkers. Zhou et al. (2024) incorporated serum neuron-specific enolase (NSE), neurofilament light chain (NfL), and S100B into the TAVREC trial protocol as candidate biomarkers intended to complement behavioral assessment. These three markers were selected because each has prior, cross-population evidence as an objective index of brain injury and outcome: NSE and S100B reflect neuronal and glial injury and have been associated with injury severity and prognosis after cardiac arrest and traumatic brain injury, whereas NfL is a sensitive marker of axonal damage that correlates with disease severity and prognosis in DoC. Because this is a study protocol, it does not yet provide outcome evidence that taVNS reduces NSE or S100B levels. At present, these biomarkers should be regarded as promising exploratory indicators whose clinical utility for monitoring taVNS response remains to be established.

### 3.5 Autonomic nervous modulation

The efficacy of taVNS is not limited to the CNS. Recent work suggests that autonomic responses measured through HRV may help characterize physiological reactivity during taVNS and may be associated with subsequent changes in consciousness level (Li et al., 2025). In particular, indices such as the low-frequency/high-frequency (LF/HF) ratio may have potential value for responder stratification. However, these findings remain early and should not yet be interpreted as establishing HRV as a routine standalone predictor for clinical decision-making. Autonomic responses may also be relevant to the broader vagal-brainstem-cortical framework discussed above. Nevertheless, the extent to which HRV changes directly index NTS-LC-ARAS engagement in DoC remains unresolved, and current evidence is insufficient to determine whether autonomic modulation is a causal mechanism, a correlated marker, or both.

## 4 Clinical application progress of taVNS in DoC

Early clinical studies of taVNS in DoC were predominantly single-center, small-sample, or non-randomized investigations, limiting the strength of inference. More recent sham-controlled trials and multicenter protocols have improved methodological rigor, but the clinical evidence base remains heterogeneous and still includes substantial uncertainty regarding effect size, durability, and responder profiles. Table 1 summarizes representative studies published from 2017 to 2026, grouped by study design and evidence level.

**Table 1:** Summary of representative studies of Transcutaneous Auricular Vagus Nerve Stimulation (taVNS) for Disorders of Consciousness (2017–2026), grouped by evidence level.

Study (author, year)	Design	*n*	Stimulation protocol (site/frequency/duration)	Outcome measures	Main findings and features	Reference
Completed clinical studies						
Gao et al. (2025)	Case	1 (UWS)	Bilateral cymba conchae + music; 25 Hz; 300 μs; 4–6 mA; 1 h/d, 3 months	CRS-R, fNIRS	CRS-R 2 → 14 (UWS → MCS+) and frontoparietal connectivity increased during a combined taVNS + patient-preferred music intervention. Single case; cannot isolate taVNS-specific efficacy.	Gao et al. (2025)
López-Rodríguez et al. (2025)	Methods/EEG assessment report	14	Auricular (cymba/cavum/tragus); 0.5–3.0 mA; 20–60 min	CRS-R, EEG (MMN/P300)	Methods paper describing taVNS with EEG assessment in a 14-patient IRENEA-Vithas cohort that overlaps with Noé et al. (2020); not counted as independent efficacy evidence	López-Rodríguez et al. (2025)
Zhou et al. (2023a)	RCT, double-blind, sham-controlled	57 completed (28 active/29 sham); 60 enrolled	Left cymba conchae; sinusoidal; 200 μs; 20 Hz; intensity 15; 30 min × 2/d, 6 d/wk.; 4 weeks	CRS-R (total + MCS/VS–UWS subgroups)	Overall CRS-R not significant (10.93 ± 4.99 vs. 9.28 ± 4.38, *p* = 0.198); positive only in the MCS subgroup (*p* = 0.035), not VS/UWS (*p* = 0.330).	Zhou et al. (2023a)
Zhou et al. (2023b)	RCT, double-blind, sham-controlled	50 MCS analyzed (60 assessed/51 randomized)	Left cymba conchae; 20 Hz; 200 μs; 30 min × 2/d, 6 d/wk.; 4 wk. + 8-wk follow-up	CRS-R, GCS, DRS, EEG, USEP, BAEP, P300	Active > sham CRS-R at week 4 (14 vs. 10, *p* = 0.023). Shares registration ChiCTR2200066629 with the companion report (same group); possible cohort overlap — not independent confirmatory evidence.	Zhou et al. (2023b)
Yifei et al. (2022)	Randomized pilot (taVNS vs. tnVNS)	12	Bilateral; 20 Hz; 30 min × 2/d; 4 weeks	CRS-R, EEG	Modulated delta/beta power; increased DMN functional connectivity.	Yifei et al. (2022)
Osińska et al. (2022)	Longitudinal case study	1 (chronic UWS)	Left; 25 Hz; daily; 6 months (home-based)	CRS-R, EEG, HRV	CRS-R rose 4/6 → 12/13 (→ MCS); increased alpha power over months.	Osińska et al. (2022)
Yu et al. (2021)	Open-label pilot	10	Bilateral (cymba + cavum conchae); 20 Hz; 30 min × 2/d; 4 weeks	ASL-fMRI	Increased cerebral blood flow in responders (RtAS) vs. non-responders (nRtAS).	Yu et al. (2021)
Noé et al. (2020)	Prospective cohort/feasibility	14	Left (tragus); 20 Hz; 30 min × 2/d; 4 weeks	CRS-R (HR/BP/ECG safety monitoring)	Significant CRS-R gain at 1-month follow-up (*p* = 0.04); 5/8 MCS improved (*p* = 0.02), mainly motor subscale; no VS/UWS improved.	Noé et al. (2020)
Hakon et al. (2020)	Feasibility trial (open-label)	5	Left; 25 Hz; 4 h/d; 8 weeks	CRS-R	Safety/feasibility of high-dose (4 h/d) stimulation in severe TBI.	Hakon et al. (2020)
Yu et al. (2017)	Case report	1	Bilateral; 20 Hz; 30 min × 2/d; 4 weeks	CRS-R, fMRI	First case report/pilot; Demonstrated DMN connectivity changes and safety.	Yu et al. (2017)
Registered/ongoing protocols (no efficacy results yet)						
Jiao et al. (2025)	Study protocol	50 planned	Bilateral; 4/20 Hz; 30 min × 2/d; 4 weeks	CRS-R, EEG	Dense–sparse paradigm; EEG-based responder prediction / indication screening	Jiao et al. (2025)
Wang et al. (2024)	RCT protocol (multicenter, stratified, double-blind)	382 planned	Bilateral; 20 Hz; 30 min × 2/d; 8 weeks	CRS-R, FOUR, NCS-R	Largest planned trial; NCS-R-guided titration uses current reduction when NCS-R ≥ 4/nociceptive responses occur, with HRV/ECG safety assessment.	Wang et al. (2024)
Zhou et al. (2024)	RCT protocol (multicenter, triple-blind)	116 planned	Left cymba conchae; 25 Hz; 300 μs; 1 mA; monophasic square; 60 min × 2/d; 4 weeks	CRS-R, GCS, FOUR, BAEP/USEP, PET/fMRI, serum NSE/NfL/S100B	Adds serum biomarkers + multimodal assessment to behavioral outcomes.	Zhou et al. (2024)
Vitello et al. (2023)	RCT protocol (double-blind, parallel)	44 planned	Bilateral; 25 Hz; 45 min/d × 5 days	CRS-R, HD-EEG	Early period (7–90 d post-injury), ICU acute-to-subacute, mixed etiology; ear-lobe sham; 3-month functional follow-up.	Vitello et al. (2023)

RCT, randomized controlled trial; UWS, unresponsive wakefulness syndrome; VS, vegetative state; MCS, minimally conscious state; CRS-R, Coma Recovery Scale–Revised; FOUR, Full Outline of UnResponsiveness; NCS-R, Nociception Coma Scale–Revised; GCS, Glasgow Coma Scale; DRS, Disability Rating Scale; EEG, electroencephalography; HD-EEG, high-density EEG; ERP, event-related potential; MMN, mismatch negativity; fNIRS, functional near-infrared spectroscopy; fMRI, functional magnetic resonance imaging; ASL, arterial spin labeling; HRV, heart rate variability; DMN, default mode network; TBI, traumatic brain injury; RtAS/nRtAS, responsive/non-responsive to auditory stimulation; tnVNS, transcutaneous non-auricular vagus nerve stimulation. BAEP, brainstem auditory evoked potential; USEP, upper-limb somatosensory evoked potential; P300, P300 event-related potential; PET, positron emission tomography; NSE, neuron-specific enolase; NfL, neurofilament light chain; S100B, S100 calcium-binding protein B.

### 4.1 Early exploration phase

In the initial phase of taVNS application, research focused on safety and preliminary efficacy exploration. Yu et al. (2017) reported the first case—a 73-year-old woman in VS/UWS 50 days after cardiopulmonary resuscitation—in whom 4 weeks of bilateral conchal taVNS was accompanied by an increase in CRS-R score and altered default mode network connectivity on fMRI; as a single case, it provided only preliminary feasibility evidence. Noé et al. (2020) subsequently studied a cohort of 14 patients (8 MCS and 6 VS/UWS) and observed greater CRS-R improvement in MCS than in VS/UWS patients. Hakon et al. (2020) reported a feasibility study in 5 patients with severe traumatic brain injury (TBI), in whom high-dose stimulation was tolerated and isolated transitions toward MCS or emergence from MCS (EMCS) were observed. Because these studies had very small samples, were single-center, and generally lacked robust control conditions, they are best interpreted as feasibility and signal-detection studies rather than as evidence establishing efficacy, since natural recovery, spontaneous fluctuation, and nonspecific effects cannot be excluded.

### 4.2 Mechanism exploration and efficacy prediction phase

The subsequent phase of research began exploring taVNS mechanisms of action and efficacy predictors, introducing functional imaging and electrophysiological assessment tools. Yu et al. (2021) in 2021 used fMRI to monitor the effects of taVNS on cerebral blood flow in DoC patients. The study enrolled 10 DoC patients (5 responsive to auditory stimulation and 5 non-responsive), and after 4 weeks of taVNS treatment, responsive patients achieved favorable outcomes while non-responsive patients had poor prognosis. Cerebral blood flow analysis showed that taVNS significantly increased blood flow in multiple brain regions of responsive patients, while non-responsive patients showed virtually no change (only slight increase in the left cerebellum). This study suggested that preserved auditory responsiveness may be associated with a greater likelihood of benefit from taVNS and proposed a possible role for salience, limbic, and interoceptive networks. Given the small sample and open-label design, these findings should be interpreted as hypothesis-generating rather than as definitive evidence for patient selection or mechanism.

### 4.3 Randomized controlled trial phase

In recent years, sham-controlled trials and multicenter protocols have improved the methodological rigor of the taVNS evidence base. Zhou et al. (2023a) conducted a randomized, double-blinded, sham-controlled study in which 60 patients with DoC were enrolled and 57 completed the intervention (28 active, 29 sham). The overall post-treatment CRS-R score did not differ significantly between the active- and sham-stimulation groups (10.93 ± 4.99 vs. 9.28 ± 4.38; *p* = 0.198). The positive signal came mainly from the MCS subgroup (14.63 ± 2.63 vs. 12.62 ± 2.50; *p* = 0.035), whereas the VS/UWS subgroup did not show a significant between-group difference (6.00 ± 2.30 vs. 5.15 ± 1.91; *p* = 0.330). Thus, the current randomized evidence should be described as a preliminary subgroup-level treatment signal rather than as validated overall efficacy. A second report by Zhou et al. (2023a) focused on patients with MCS and reported higher CRS-R scores in the active group at week 4; however, because both 2023 reports were registered under ChiCTR2200066629 and came from the same research group, potential cohort or reporting overlap cannot be excluded. We therefore present both reports transparently in Table 1 but do not treat them as two fully independent sources of confirmatory efficacy evidence. In addition, the “sham” condition used in some studies may not be physiologically inert; at the same time, any sensory mismatch between active and sham stimulation could compromise blinding, so the direction of the resulting bias may differ across study settings and should not be assumed to lead uniformly to an underestimation of taVNS efficacy.

Several larger and multicenter studies are now underway or available as published protocols. Zhou et al. (2024) published the first multicenter, triple-blind RCT protocol for pDoC patients in 2024, planning to recruit 116 patients with a multimodal assessment system including behavioral scales, neurophysiological indicators, neuroimaging, and serum biomarkers, with the primary outcome being consciousness level improvement based on CRS-R at week 4. Jiao et al. (2025) published a prospective exploratory clinical trial protocol in 2025, planning to enroll 50 pDoC patients for 4 weeks of taVNS treatment, assessed through pre- and post-treatment 30-min resting-state EEG and CRS-R scoring, with follow-up to 12 months post-treatment. This study aims to explore candidate EEG features that may be associated with response to taVNS through power spectral and functional connectivity analyses, rather than to establish an already validated indication-screening tool. Wang et al. (2024) designed a multicenter double-blind RCT protocol in 2024 planning to recruit 382 prolonged DoC patients with bilateral synchronous taVNS treatment. This protocol uses NCS-R scores to guide individualized stimulation-intensity reduction when nociceptive responses are observed and comprehensively assesses cardiac autonomic nervous function safety through HRV analysis.

Taken together, these protocols indicate that the field is moving toward larger, more standardized, and multimodal trials. Until their results are available, however, protocol papers should be presented as future evidence generators rather than as evidence that taVNS is already effective for pDoC.

### 4.4 Innovative exploration

Several innovative case studies have explored the possibilities of extended treatment duration and combination with other interventions, providing new perspectives for taVNS clinical application. Gao et al. (2025) reported a long-term taVNS treatment case study in 2025, applying bilateral taVNS for 3 months to a UWS patient (hemorrhagic stroke) 50 days post-onset, innovatively using fNIRS to monitor dynamic changes in frontoparietal network functional connectivity. Results showed the patient’s CRS-R score increased from 2 to 14, with the diagnosis transitioning from UWS to MCS+, accompanied by significantly enhanced frontoparietal functional connectivity. This case illustrates the feasibility of using fNIRS to track longitudinal network changes during prolonged taVNS-based rehabilitation. Because the intervention combined taVNS with music stimulation in a single patient, it cannot determine the specific contribution of each component, but it does support further investigation of multimodal rehabilitation paradigms.

Across the available small studies, patients in MCS appear to show more frequent behavioral improvement after taVNS than patients in VS/UWS, suggesting that preserved residual neural function may be associated with a greater likelihood of response. However, this should be regarded as a preliminary subgroup-level signal rather than a confirmed treatment-effect modifier, particularly because some reports may overlap and because baseline severity, etiology, time since injury, spontaneous recovery, and residual network integrity may confound apparent subgroup differences. The case study by Gao et al. (2025) raises the possibility that longer treatment duration may benefit selected UWS patients. This observation is hypothesis-generating and should not yet be translated into general clinical recommendations without controlled validation.

## 5 Stimulation parameter optimization and safety assessment

### 5.1 Stimulation parameters

In the clinical practice of taVNS treatment for DoC patients, parameter selection exhibits significant individualized characteristics and inter-study heterogeneity. Given the diversity of etiology, disease stage, and residual neural function in DoC, parameter settings are likely to require individualization. At present, however, the empirical basis for precision parameter selection remains limited, and most recommendations should be regarded as provisional rather than definitive. Based on comprehensive analysis of existing literature, commonly used taVNS treatment parameters include: stimulation frequency concentrated in the 20–25 Hz range, pulse width typically 200–500 μs, stimulation intensity ranging from 0.5–6.0 mA (mostly individually adjusted based on perceptual threshold), and treatment duration varying from single interventions to several weeks of continuous treatment, with the most common protocol being 30 min per session, twice daily. Regarding stimulation targets, the primary choices are the cymba conchae or medial tragus region, with most studies favoring left auricular stimulation to avoid potential cardiac side effects, while some studies explore the feasibility of bilateral synchronous stimulation.

#### 5.1.1 Waveform and frequency

In the taVNS literature for DoC treatment, stimulation frequency is primarily concentrated in the 20–25 Hz range. Multiple studies have adopted a frequency of 20 Hz (Yu et al., 2017; Yu et al., 2021; Zhou et al., 2023a,b; Yifei et al., 2022), while others have employed a slightly higher frequency of 25 Hz (Noé et al., 2020; Hakon et al., 2020; Osińska et al., 2022; Vitello et al., 2023). This clustering suggests that 20–25 Hz is a commonly used empirical range in current studies, but it does not by itself establish this range as the optimal therapeutic window for DoC. Waveform selection also varies: Osińska et al. (2022) used monophasic square waves, while Noé et al. (2020) used sinusoidal waveforms. Notably, a considerable number of studies did not explicitly specify the waveform type, presumably using the default waveform of their devices (Yu et al., 2017; Hakon et al., 2020; Yu et al., 2021; Zhou et al., 2023a,b; Yifei et al., 2022; Vitello et al., 2023). This inconsistency and information gap in reporting highlights the necessity of establishing standardized reporting norms to comprehensively understand the mechanistic differences of various waveforms in taVNS treatment. Waveform characteristics affect fiber recruitment, tolerability, and autonomic responses, thereby influencing stimulation effects. In this context, appropriate pulse width selection can more effectively achieve selective activation of target fibers, optimizing stimulation outcomes.

To mitigate potential neural habituation effects during long-term treatment, novel waveform designs have emerged. Unlike the conventional monophasic square wave used in the TAVREC study (Zhou et al., 2024), Jiao et al. (2025) introduced a “dense-sparse wave” (alternating 4 Hz/20 Hz) in their latest protocol. This dynamically modulated stimulation pattern is intended to reduce habituation during prolonged stimulation, but its comparative advantage over conventional waveforms remains hypothetical until tested in completed clinical studies.

#### 5.1.2 Intensity

Determining stimulation intensity for non-communicative DoC patients has been a persistent challenge, and early studies mostly relied on researchers’ subjective judgment or fixed subthreshold intensities. More recent protocols have explored the Nociception Coma Scale-Revised (NCS-R) as an investigational aid for intensity titration (Wang et al., 2024; Gao et al., 2025). Importantly, the purpose of this procedure is not to infer effective vagal-fiber recruitment from the NCS-R score; the NCS-R reflects behavioral responses to nociceptive stimulation, and a score ≥4 should trigger a reduction—not an increase—in current. In the multicenter protocol by Wang et al. (2024), when the NCS-R score is ≥4 the current is reduced in 0.5-mA steps and reassessed until nociceptive responses are no longer observed, and patients who still show nociceptive responses at the minimum intensity are excluded. Similarly, in the prolonged case reported by Gao et al. (2025), the current was reduced by 0.2 mA and reassessed whenever the NCS-R score exceeded 4. This approach may improve tolerability and standardization relative to fixed-dose stimulation, but it remains investigational and has not been shown to be superior to other methods of determining stimulation intensity, nor has an NCS-R-defined threshold been validated as a marker of effective neural activation.

#### 5.1.3 Stimulation site

The conventional view holds that stimulation should be limited to the left vagus nerve to avoid potential cardiac side effects (such as bradycardia). However, the anatomical structure and physiological functional connections of the auricular branch of the vagus nerve may differ from those of the cervical vagus nerve (Elamin et al., 2023). Recent multicenter RCT protocols have begun exploring bilateral synchronous stimulation strategies, with their safety and potential augmentation value still requiring further validation following trial result publication. Wang et al.’s, 2024 large RCT adopted a bilateral cymba conchae stimulation protocol, with the theoretical rationale that bilateral input may recruit a broader brainstem reticular activating system (ARAS). This contrasts with unilateral stimulation protocols such as TAVREC and reflects growing interest in whether broader afferent recruitment may enhance efficacy. For now, bilateral stimulation should still be regarded as an exploratory strategy pending outcome and safety data from completed trials.

### 5.2 Safety and adverse events

Overall, taVNS demonstrates good safety and tolerability in DoC patients, and it has been studied across several conditions, including DoC, depression, epilepsy, and migraine. Given that treatment options for DoC patients may be limited, the low risk of serious complications associated with taVNS makes it an attractive treatment option (Dong and Feng, 2018). In clinical studies, taVNS has shown good tolerability and compliance, with most DoC patients completing treatment without major adverse events (Yu et al., 2017; Yu et al., 2021; Zhou et al., 2023a,b). Side effects of taVNS are typically mild and transient, including skin irritation, itching, or redness at the electrode placement site, as well as occasional dizziness, headache, or nausea (Zhou et al., 2023a,b).

Historically, right-sided vagus nerve stimulation has been approached cautiously due to its extensive innervation of the sinoatrial node, but auricular stimulation differs from cervical vagus nerve stimulation in anatomy and fiber recruitment. Wang et al.’s (2024) RCT protocol employs bilateral synchronous taVNS with electrocardiographic monitoring systems to assess potential cardiac risks. Common side effects remain limited to mild and transient skin irritation or redness at electrode contact sites, with no systemic adverse reactions observed. Current conclusions regarding the safety of bilateral stimulation are based mainly on preliminary reports and protocol designs and therefore require confirmation from adequately powered completed trials.

Safety assessment has evolved from passive “adverse event recording” to active “physiological indicator monitoring.” Current high-quality clinical trials require continuous HRV and electrocardiographic monitoring during treatment. Notably, research published in 2025 suggests that autonomic responses (such as changes in the LF/HF ratio) may serve not only as safety signals but also as candidate efficacy-related markers (Li et al., 2025). This raises the possibility that moderate autonomic engagement may be associated with therapeutic response. Whether it reflects a safety signal, a mechanistic mediator, a treatment correlate, or some combination of these remains to be clarified.

Evidence from invasive VNS and animal studies suggests that splenic pathways may contribute to vagally mediated anti-inflammatory effects (Huston et al., 2006; Huston et al., 2009; Gigliotti et al., 2013; Ji et al., 2014), but the translational relevance of this mechanism to taVNS in DoC remains indirect. From a safety perspective, patients with arrhythmia, implanted cardiac devices, uncontrolled epilepsy, severe cardiopulmonary instability, or other major contraindications should be carefully evaluated before taVNS is initiated (Vaseghi et al., 2017; Yuan et al., 2017). Individualized treatment plans should consider etiology, disease stage, baseline consciousness level, autonomic status, medication exposure, and the feasibility of monitoring during stimulation.

## 6 Discussion

This review examined recent advances in taVNS for DoC from four perspectives: mechanisms, multimodal neurophysiological evidence, clinical efficacy, and stimulation parameter optimization with safety assessment. Overall, current evidence supports taVNS as a promising adjunctive neuromodulation strategy for DoC, particularly in patients with relatively preserved residual function such as those in MCS. However, the field still faces major challenges in mechanism validation, evidence integration, parameter standardization, and long-term efficacy assessment.

From a mechanistic perspective, the vagal-cortical pathway model proposed by Briand et al. (2020) provides a useful theoretical framework for understanding possible taVNS effects, whereby ABVN stimulation may engage brainstem nuclei such as the NTS, LC, and DRN and subsequently influence cortical networks. Frequency-dependent effects on brain network connectivity reported by Rong et al. (2016) support the broader idea that stimulation parameters may shape network modulation, but this evidence comes from non-DoC populations and should be treated as indirect. Likewise, fNIRS observations by Gao et al. (2025) and HRV findings by Li et al. (2025) provide clinically relevant associations between taVNS-based interventions, network/autonomic changes, and consciousness-related outcomes, but they do not yet establish a direct causal chain from peripheral stimulation to network reorganization and consciousness recovery in DoC.

From the neurophysiological evidence perspective, a central limitation of the current taVNS literature in DoC is the lack of synchronized multimodal evidence within the same cohorts. fNIRS, fMRI, EEG, PET, HRV, and peripheral biomarker measures each address different levels of the proposed mechanism, but most findings come from different studies, patient populations, and time points, making causal interpretation difficult. Bedside-accessible modalities such as fNIRS and EEG have accumulated comparatively more data, whereas fMRI and PET evidence capable of characterizing subcortical structures and global metabolism remains sparse. A key priority is therefore to move beyond isolated single-modality observations and toward synchronized multimodal monitoring protocols that integrate central and peripheral indicators within the same treatment cycle and patient cohort. Such designs may help move the literature from descriptive association toward more informative mechanistic inference.

From a clinical efficacy perspective, patients in MCS appear to respond more often than those in VS/UWS; this is best regarded as a preliminary subgroup signal rather than an established treatment-effect modifier, because baseline severity, etiology, time since injury, the probability of spontaneous recovery, and residual network integrity may all act as confounders. Understanding the poorer apparent response in VS/UWS, and whether optimizing parameters or extending treatment duration can improve outcomes in this population, remains an important question. The case study by Gao et al. (2025) raises the possibility that prolonged treatment may benefit selected UWS patients, but current evidence is insufficient to support broad treatment-duration recommendations for VS/UWS on the basis of single-case experience.

Consistent with this cautious interpretation, a 2026 individual-patient-data meta-analysis of vagus nerve stimulation (invasive and non-invasive) for DoC, pooling 10 studies and 112 patients, reported a modest mean CRS-R improvement of 2.78 points (95% CI 1.62–3.94), with 40.2% of patients reaching the minimal clinically important difference (≥3 points) and greater improvement in MCS than in coma or VS/UWS; the authors explicitly called for adequately powered randomized trials of both invasive and non-invasive VNS (Zhang et al., 2026).

Stimulation parameter optimization represents both a focus and challenge of current research. Existing studies show considerable variability in frequency, pulse width, intensity, and treatment duration parameters, making it difficult to compare and synthesize results. Although 20–25 Hz frequency is widely adopted, this is primarily based on FDA-approved VNS parameters rather than systematic optimization specific to taVNS and DoC. The core challenge of parameter optimization lies in the inability of DoC patients to provide subjective feedback, making traditional sensory threshold-based parameter adjustment methods difficult to apply. Currently, ongoing exploratory studies employ innovative designs to evaluate the immediate effects of different frequencies, which will provide a foundation for individualized treatment. Meanwhile, we must recognize that optimal parameters may vary according to patient etiology, disease course, baseline consciousness level, and brain injury location; a single standardized protocol may not meet the needs of all patients. Therefore, future research should aim to establish individualized parameter optimization protocols based on patient characteristics, requiring both large-scale clinical trial support and in-depth understanding of taVNS mechanisms of action.

Despite these advances, the available taVNS evidence for DoC remains limited by small samples, single-center designs, case-report evidence, protocol heterogeneity, and limited follow-up. Stimulation frequency, intensity, duration, waveform, and electrode placement vary substantially across studies, which makes comparison and synthesis difficult. Many reports focus on short-term behavioral or physiological changes, whereas durability, functional outcomes, and cumulative effects of repeated or prolonged taVNS remain uncertain. In addition, proposed mechanisms remain incompletely validated, limiting our ability to explain individual differences in response or to optimize treatment protocols.

Future research needs to advance on multiple levels. First, large-sample, multicenter, rigorously designed RCTs remain crucial. These trials should employ standardized assessment tools and treatment protocols to provide stronger evidence for taVNS efficacy in DoC patients. Trial design should account for patient heterogeneity by stratifying for etiology, disease course, and consciousness level so that subgroup signals can be tested rather than inferred retrospectively. Second, future research should clarify the neural pathways and molecular mechanisms underlying taVNS effects through synchronized multimodal monitoring within the same patient cohorts. Combining neuroimaging, neurophysiological assessment, autonomic measures, and candidate serum biomarkers may help move the field from descriptive associations toward more informative mechanistic inference. Third, metabolomic and fluid-biomarker technologies may become important exploratory tools for responder stratification, but they should be validated against prespecified behavioral and long-term functional outcomes before entering routine decision-making. Fourth, protocols such as that proposed by Jiao et al. (2025) reflect growing interest in EEG-informed responder stratification and may represent an important step toward more individualized taVNS trials. Future clinical pathways should avoid a “one-size-fits-all” approach and should test whether baseline residual network status measured by EEG, fNIRS, fMRI, PET, HRV, or combined approaches can serve as candidate predictors of response. Finally, combined interventions—for example, taVNS together with hyperbaric oxygen therapy, transcranial direct current stimulation, or conventional pharmacotherapy—may ultimately help overcome therapeutic barriers in refractory cases, but they must be evaluated with designs capable of separating the specific contribution of taVNS from that of the other rehabilitation or neuromodulation components (Liu et al., 2025).

## 7 Conclusion

In summary, taVNS is a practical non-invasive neuromodulation strategy that may influence consciousness-related circuits through a peripheral-vagus–brainstem–cortical framework. Current evidence supports its promise as an adjunctive intervention for DoC, particularly in selected patients with MCS, but the mechanistic model remains incompletely validated and the clinical signal is still preliminary. Recent studies have expanded the field from predominantly exploratory work toward more clinically oriented human research, including sham-controlled trials, multicenter protocols, objective titration strategies, and candidate physiological biomarkers. Nevertheless, insufficient multimodal evidence integration, limited long-term efficacy data, and gaps in individualized parameter optimization remain major barriers to standardized clinical application. At present, taVNS should be viewed as a promising but provisional adjunctive intervention rather than a standard treatment, pending stronger evidence on efficacy, durability, optimal parameters, safety in larger cohorts, and responder selection.
